# Assessment of Mandibular Bone Architecture in Patients with Endocrine Disorders Using Fractal Dimension and Histogram Analysis

**DOI:** 10.3390/tomography11060070

**Published:** 2025-06-18

**Authors:** Elif Yıldızer, Saliha Kubra Sari, Fatih Peker, Ali Riza Erdogan, Kevser Sancak, Sinan Yasin Ertem

**Affiliations:** 1 Department of Oral and Maxillofacial Radiology, Faculty of Dentistry, Ankara Yildirim Beyazit University, 06010 Ankara, Turkey; elifyildizer@aybu.edu.tr (E.Y.); salihakubra.sari@aybu.edu.tr (S.K.S.); fatihpeker@aybu.edu.tr (F.P.); 2 Faculty of Medicine, Hoca Ahmet Yesevi International Turk-Kazak University, Turkestan 161200, Kazakhstan; aliriza.erdogan@ayu.edu.kz; 3 Department of Oral and Maxillofacial Surgery, Faculty of Dentistry, Ankara Yildirim Beyazit University, 06010 Ankara, Turkey; ksancak@aybu.edu.tr

**Keywords:** fractal dimension analysis, mandibular bone microarchitecture, endocrine disorders, panoramic radiography

## Abstract

Objective: Endocrine disorders, including diabetes mellitus and thyroid dysfunctions, can significantly impact bone metabolism and structure. This study aimed to assess mandibular trabecular architecture in patients with type 1 diabetes mellitus (T1DM), type 2 diabetes mellitus (T2DM), hyperthyroidism, and hypothyroidism using fractal dimension (FD) and histogram analyses (HA), comparing the findings with a healthy control group. Methods: This retrospective study analyzed panoramic radiographs from 200 individuals, comprising 40 patients in each of the four endocrine disorder groups and 40 healthy controls. Fractal dimension and histogram-based pixel intensity analyses were conducted using ImageJ™ (version 1.53) software. Four standardized regions of interest (ROI) were evaluated on the right mandible, and statistical comparisons were conducted across groups using one-way analysis of variance (ANOVA), *t*-test, Mann–Whitney U, and Spearman correlation analyses. Results: Age and gender distributions did not differ significantly between groups. FD analysis revealed a significant reduction at ROI1 in the hyperthyroidism group compared to controls (*p* = 0.018); however, no other significant FD differences were observed among the remaining groups or ROIs. A significant positive correlation was found between FD and histogram values at ROI1 and ROI2 (*p* < 0.001), while pixel intensity values did not differ significantly across groups in any ROI. Conclusion: Although no significant differences were found in diabetic groups, the decreased FD in hyperthyroid patients suggests that FD analysis may be a useful non-invasive method to detect subtle bone alterations. Further research with larger sample sizes and comprehensive biochemical analyses are needed to confirm these findings.

## 1. Introduction

Endocrine system diseases arise from hormonal imbalances and can impact multiple organ systems [1]. Among these, diabetes, hyperthyroidism, and hypothyroidism are key examples. Hormones play a crucial role in regulating various tissues and organs, including the cardiovascular, respiratory, gastrointestinal, neurological, and musculoskeletal systems, with a significant influence on bone health [1,2]. As a primary target of hormonal activity, bone undergoes remodeling processes that are directly affected by hormonal fluctuations [2].

Osteoporosis is a condition marked by reduced bone mass and microarchitectural deterioration, increasing susceptibility to fractures. Endocrine disorders are a major cause of secondary osteoporosis [3]. Diabetes, in particular, negatively affects bone metabolism, raising fracture risk [4]. Type 1 diabetes mellitus (T1DM) results from insulin deficiency, while type 2 diabetes mellitus (T2DM) is characterized by insulin resistance often accompanied by an insufficient compensatory insulin secretion [5]. Both conditions impair bone health by disrupting bone formation, resorption, collagen synthesis, inflammatory processes, and calcium metabolism [4]. Despite differing effects on bone mineral density (BMD), both T1DM and T2DM are associated with an increased risk of fractures [6].

Thyroid hormones are essential for skeletal development and bone homeostasis [7]. Hyperthyroidism, characterized by excessive thyroid hormone production, is commonly linked to decreased BMD and a higher risk of fractures [8]. Hypothyroidism can be either subclinical—where thyroid stimulating hormone (TSH) levels are elevated but free T4 remains normal—or overt, with both high TSH and low free T4 levels [9]. In both cases, thyroid dysfunction can negatively impact bone health [8].

The measurement of BMD using dual-energy X-ray absorptiometry (DXA) is the gold-standard method for diagnosing osteoporosis [10]. However, BMD alone is insufficient to fully assess fracture risk, as bone fragility is also influenced by factors such as bone remodeling activity, trabecular microarchitecture, mineral distribution, and skeletal geometry [11]. Fractal analysis (FA) is a valuable technique for evaluating bone microarchitecture. Since trabecular bone exhibits fractal properties, FA is widely used to analyze its structural complexity [12,13]. This method quantifies bone structure using fractal dimension (FD) [14]. The box-counting algorithm, a commonly applied FA technique, assesses the fractal dimension of the trabecular bone and bone marrow interface and an increased box-counting value reflects greater structural complexity [15]. Studies have demonstrated the effectiveness of FA in detecting structural bone changes [12,16]. Pixel intensity (PI) analysis is another imaging technique used to evaluate bone tissue in digital images [17]. By measuring grayscale variations, PI analysis provides insight into bone density, often utilizing histogram analysis for interpretation [17,18].

Although extensive research has explored the impact of endocrine diseases on overall bone health [6,8], studies focusing on the maxillofacial region remain limited [19,20]. In particular, the effects of thyroid disorders on jawbone structure are not well understood, and there is currently no research evaluating the impact of thyroid diseases on the mandible using both fractal and histogram analyses. This study aims to assess the impact of T1DM, T2DM, hypothyroidism, and hyperthyroidism on mandibular trabecular architecture using fractal dimension and histogram analysis, comparing findings with those from healthy individuals.

## 2. Materials and Methods

### 2.1. Ethics Approval

This retrospective study was conducted using panoramic radiographs from the database of the Department of Oral and Maxillofacial Radiology, Faculty of Dentistry, Ankara Yildirim Beyazit University, collected between 2022 and 2023. The study adhered to the ethical principles outlined in the Declaration of Helsinki. Ethical approval was granted by the Health Sciences Ethics Committee of Ankara Yildirim Beyazit University (Decision No: 339/07).

### 2.2. Patient Selection

The required sample size for this retrospective study was determined using the G*Power (ver. 3.1.9.7) statistical software. The study included five groups: four disease subgroups (hyperthyroidism, hypothyroidism, type 1 diabetes mellitus, and type 2 diabetes mellitus) and one control group. Using an independent samples *t*-test design, the parameters were set as follows: Statistical power: 0.80, Effect size: 0.25 (based on the F-test effect size range), Type I error (α): 0.05. Based on these calculations, a total of 200 panoramic radiographs were analyzed, with at least 40 samples per group.

Inclusion criteria for the patient group: Patients diagnosed with type 1 or type 2 diabetes mellitus, hyperthyroidism, or hypothyroidism, based on medical history and anamnesis records retrieved from the hospital information system. All patients were under medical control, had no other systemic diseases, and had not been diagnosed with osteoporosis. Panoramic radiographs had to meet acceptable image quality standards.

Inclusion criteria for the control group: Individuals with no known systemic diseases affecting bone metabolism and not taking any medications that could influence bone health. Panoramic radiographs also needed to have sufficient image quality.

Exclusion criteria (both groups): Patients with incomplete medical or anamnesis records in the hospital system; any structural defects or abnormalities (e.g., surgical alterations, fractures, cysts or tumours) in the regions of interest; unclear anatomical landmarks in the target areas on the panoramic radiograph; poor image quality; presence of artifacts or ghost images that could compromise analysis.

### 2.3. Panoramic Radiography Protocol

All panoramic radiographs were acquired using a digital dental panoramic device (Planmeca, Helsinki, Finland) under standardized conditions: Exposure time: 15.8 s, voltage: 64–68 kVp, current: 6.3–10 mA. To ensure consistency, patients were positioned according to the manufacturer’s guidelines, maintaining the Frankfort plane parallel to the ground with light beam markers aligned appropriately.

### 2.4. Fractal Dimension and Pixel Intensity Analysis

Before analysis, panoramic radiographs were saved in high-resolution Tagged Image File Format (TIFF) and processed using ImageJ™ software (version 1.53, National Institutes of Health, Bethesda, MD, USA) [21]. Fractal dimension and histogram analyses were performed using this software.

Square regions of interest (ROIs) of 64 × 64 pixels were selected owing to their frequent use in previous studies [13]. Four square ROIs were then placed on the right side of the mandible for fractal dimension and histogram-based pixel intensity analyses performed on dental panoramic radiographs. ROI1 was defined at the geometric center of the area between the mandibular notch and the mandibular foramen. ROI2 was positioned in the supracortical region superior to the mandibular angle. ROI3 was placed distal to the apex of the second premolar, carefully avoiding the mandibular canal’s cortical borders, periodontal ligament space, lamina dura, and root apices. ROI4 was located anterior to the mental foramen, excluding adjacent anatomical structures.

**Figure 1:**
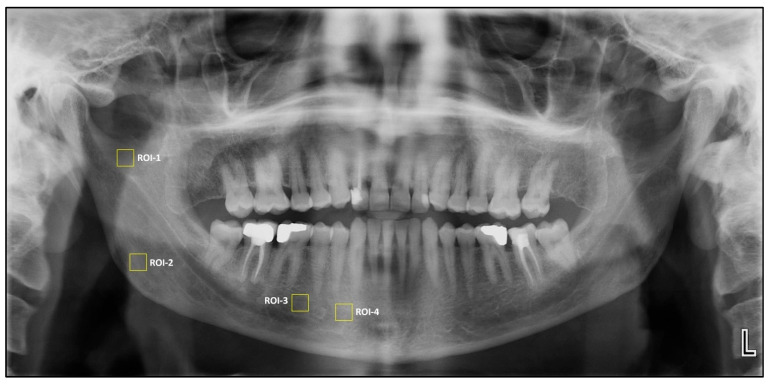
Selection of region of interest (ROIs).

**Figure 2:**
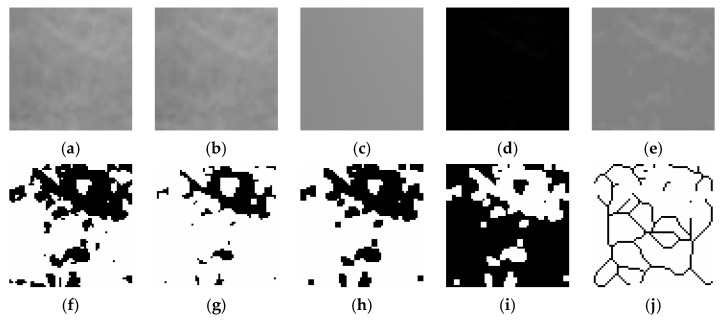
Steps of fractal dimension analysis. (**a**), The selected ROI from panoramic radiographs was cropped and (**b**), duplicated. (**c**), Gaussian blur filter was applied on duplicated image. (**d**), The blurred image was subtracted from the original image. (**e**), Addition of a gray value of 128 to each pixel location. (**f**), Binarization (**g**), Erosion (**h**), Dilation (**i**), Inversion (**j**), The skeletonized image was used for fractal dimension analysis.

**Figure 3:**
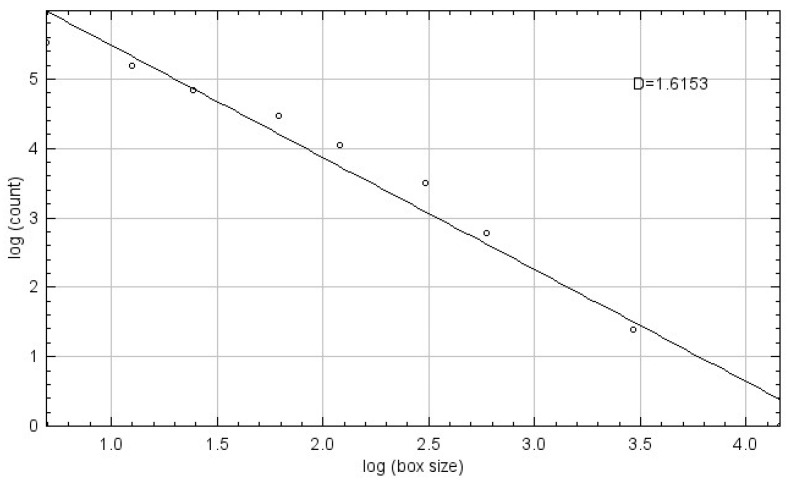
Fractal Dimension Calculation Using the Box-Counting Method.

Fractal dimension (FD) was calculated using the box-counting method proposed by White and Rudolph [22].The procedure included: selection of 64 × 64-pixel ROIs, cropping, duplication, application of Gaussian blur (sigma = 35) filter to minimize brightness variations due to overlying soft tissues and bone thickness differences, subtraction of the blurred image from the original image, discrimination of bone marrow spaces and trabeculae by adding 128 gray values to each pixel location, application of binarization, erosion, dilation, inversion, and skeletonization, and calculation of the fractal dimension (Figure 1, Figure 2 and Figure 3).

**Figure 4:**
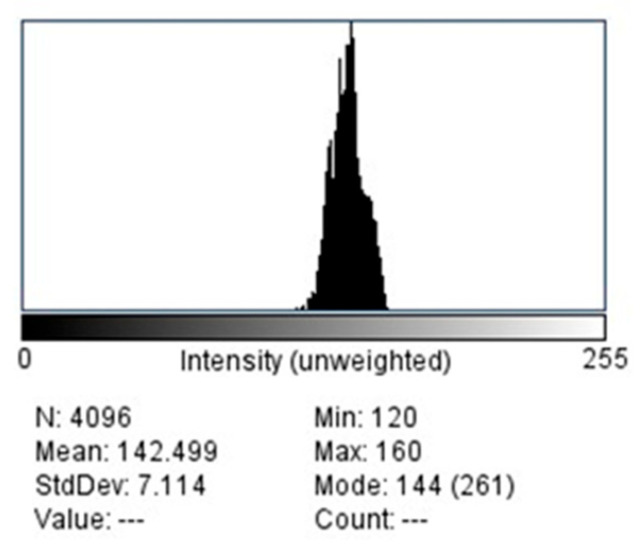
Histogram of selected ROI.

Mean pixel intensity (PI) values for each ROI were obtained using the histogram tool in ImageJ™ software (version 1.53, National Institutes of Health, Bethesda, MD, USA) (Figure 4). This analysis provided quantitative information on bone density based on grayscale variations.

### 2.5. Statistical Analysis

All statistical analyses were performed using IBM SPSS Statistics for Windows, Version 30.0 (IBM Corp., Armonk, NY, USA). All image assessments were conducted by a single experienced dentomaxillofacial radiologist (EY) with specialized training in the field. To evaluate intra-observer reliability, twenty panoramic radiographs were randomly selected and reanalyzed two weeks after the initial evaluation. This allowed for the calculation of intra-observer correlation coefficients across the four anatomical sites, using a 95% confidence interval to ensure measurement consistency and reproducibility.

Descriptive statistics were calculated for all variables. The distribution of gender across the five study groups—hyperthyroidism, hypothyroidism, type 1 diabetes mellitus (T1DM), type 2 diabetes mellitus (T2DM), and control group—was assessed using the Chi-square test. Age-related data were analyzed using one-way analysis of variance (ANOVA) to compare the mean ages between the study groups. The normality of data distribution was assessed using the Shapiro–Wilk test. For variables that did not follow a normal distribution, comparisons between groups were conducted using the non-parametric Mann–Whitney U test and variables that were normally distributed were analyzed using the independent samples *t*-test. Fractal dimension and histogram analysis values were compared between patients with hyperthyroidism, hypothyroidism, T1DM and T2DM and control group across four regions of interest (ROIs). The relationship between fractal dimension and histogram values was evaluated using Spearman’s rank correlation coefficient. A *p*-value less than 0.05 was considered statistically significant.

## 3. Results

This retrospective study included panoramic radiographs of 200 individuals, including 40 patients with type 1 diabetes mellitus, 40 patients with type 2 diabetes mellitus, 40 patients with hyperthyroidism, 40 patients with hypothyroidism, and 40 individuals from the control group.

The consistency of repeated measurements was evaluated using intra-observer correlation analysis, with statistical significance set at *p* < 0.05. No significant discrepancies were observed between the initial and repeated assessments. The reliability scores for each region of interest confirmed a high level of agreement: ROI1 (0.85), ROI2 (0.80), ROI3 (0.79), and ROI4 (0.81).

### 3.1. Comparisons of Age and Gender Variables Between the Subgroups

Table 1 shows the age and gender comparisons of the patient groups and the control group. No statistically significant difference was found between the disease groups (hyperthyroidism: 50.83 ± 8.1, hypothyroidism: 50.78 ± 8.6, type 1 diabetes mellitus: 50.85 ± 13.5, type 2 diabetes mellitus: 50.78 ± 9.0) and the control group (50.73 ± 9.9) in terms of mean age (*p* > 0.05). The number of female individuals in the disease groups and the control group (hyperthyroidism *n* = 31, hypothyroidism *n* = 34, type 1 diabetes mellitus *n* = 27, type 2 diabetes mellitus *n* = 31, control group *n* = 31) was higher than the number of male individuals (hyperthyroidism *n* = 9, hypothyroidism *n* = 6, type 1 diabetes *n* = 13, type 2 diabetes mellitus *n* = 9, control group *n* = 9). No statistically significant difference was found between the groups in terms of gender distribution (*p* > 0.05). There were no statistically significant differences in age or gender distribution between the control and disease groups, indicating that the groups were comparable in terms of demographic characteristics.

### 3.2. Fractal Dimension Analysis

In the hyperthyroidism group, a statistically significant decrease in fractal dimension was observed at ROI1 compared to the control group (*p* = 0.018), with mean values of 1.406 ± 0.160 and 1.478 ± 0.157, respectively. No significant differences were found at ROI2 (1.400 ± 0.152 vs. 1.461 ± 0.117, *p* > 0.05), ROI3 (1.510 ± 0.131 vs. 1.526 ± 0.105, *p* > 0.05), or ROI4 (1.547 ± 0.057 vs. 1.541 ± 0.061, *p* > 0.05) (Table 2).

For the hypothyroidism group, no statistically significant differences were detected across any ROI when compared to controls. The respective means and standard deviations were as follows: ROI1 (1.453 ± 0.158 vs. 1.478 ± 0.157, *p* > 0.05), ROI2 (1.453 ± 0.135 vs. 1.461 ± 0.117, *p* > 0.05), ROI3 (1.526 ± 0.070 vs. 1.526 ± 0.105, *p* > 0.05), and ROI4 (1.554 ± 0.060 vs. 1.541 ± 0.061, *p* > 0.05) (Table 2).

In patients with type 1 diabetes mellitus, fractal dimension values did not significantly differ from those of the control group in any ROI: ROI1 (1.498 ± 0.150 vs. 1.478 ± 0.157, *p* > 0.05), ROI2 (1.454 ± 0.131 vs. 1.461 ± 0.117, *p* > 0.05), ROI3 (1.533 ± 0.087 vs. 1.526 ± 0.105, *p* > 0.05), and ROI4 (1.521 ± 0.087 vs. 1.541 ± 0.061, *p* > 0.05) (Table 2).

Similarly, the type 2 diabetes group also showed no significant difference compared to controls in any ROI: ROI1 (1.438 ± 0.180 vs. 1.478 ± 0.157, *p* > 0.05), ROI2 (1.453 ± 0.160 vs. 1.461 ± 0.117, *p* > 0.05), ROI3 (1.545 ± 0.062 vs. 1.526 ± 0.105, *p* > 0.05), and ROI4 (1.543 ± 0.052 vs. 1.541 ± 0.061, *p* > 0.05) (Table 2).

### 3.3. Histogram-Based Pixel Intensity Analysis

In the histogram analysis, the hyperthyroidism group showed no statistically significant differences from the control group in any ROI: ROI1 (95.988 ± 27.529 vs. 108.063 ± 32.056, *p* > 0.05), ROI2 (76.368 ± 29.770 vs. 79.843 ± 30.411, *p* > 0.05), ROI3 (89.683 ± 18.830 vs. 86.231 ± 18.349, *p* > 0.05), and ROI4 (116.103 ± 21.520 vs. 111.702 ± 18.459, *p* > 0.05) (Table 3).

Similarly, in the hypothyroidism group, none of the ROIs showed significant differences compared to the control group: ROI1 (111.134 ± 29.653 vs. 108.063 ± 32.056, *p* > 0.05), ROI2 (86.448 ± 32.121 vs. 79.843 ± 30.411, *p* > 0.05), ROI3 (89.924 ± 16.307 vs. 86.231 ± 18.349, *p* > 0.05), and ROI4 (107.222 ± 19.790 vs. 111.702 ± 18.459, *p* > 0.05) (Table 3).

In the type 1 diabetes mellitus group, histogram values also did not differ significantly from the control group across all ROIs: ROI1 (109.568 ± 28.771 vs. 108.063 ± 32.056, *p* > 0.05), ROI2 (82.291 ± 27.352 vs. 79.843 ± 30.411, *p* > 0.05), ROI3 (91.934 ± 18.693 vs. 86.231 ± 18.349, *p* > 0.05), and ROI4 (113.142 ± 24.282 vs. 111.702 ± 18.459, *p* > 0.05) (Table 3).

The type 2 diabetes group showed similar findings: ROI1 (117.618 ± 29.570 vs. 108.063 ± 32.056, *p* > 0.05), ROI2 (89.150 ± 29.037 vs. 79.843 ± 30.411, *p* > 0.05), ROI3 (93.185 ± 18.584 vs. 86.231 ± 18.349, *p* > 0.05), and ROI4 (107.877 ± 21.399 vs. 111.702 ± 18.459, *p* > 0.05) (Table 3).

### 3.4. Correlation Between Fractal Dimension and Histogram Analysis Values

A statistically significant positive correlation was found between fractal dimension and histogram analysis values at ROI1 (Spearman’s r = 0.286, *p* < 0.001) and ROI2 (r = 0.260, *p* < 0.001). However, no significant correlations were observed at ROI3 (r = 0.110, *p* > 0.05) or ROI4 (r = 0.117, *p* > 0.05) (Table 4).

## 4. Discussion

Bone health plays a critical role in dental practice and plays a decisive role in various clinical applications. In implant dentistry, sufficient bone volume and quality are essential for stability and long-term success [23]. Similarly, in periodontal diseases, alveolar bone loss reflects the severity of periodontal destruction and may provide critical diagnostic information to guide treatment strategies [14]. Orthodontic interventions rely on bone remodeling processes, making bone structure a key determinant of both treatment duration and tooth movement efficiency [24]. In this context, the condition of the supporting bone influences diagnosis, treatment planning, and clinical outcomes across nearly all fields of dentistry. Given the fundamental role of bone health in clinical dentistry, including implantology, periodontology, and orthodontics, understanding how systemic diseases such as diabetes and thyroid dysfunction affect jawbone microarchitecture is essential.

This study is among the few that have investigated mandibular bone structure in patients with hyperthyroidism, hypothyroidism, type 1 diabetes mellitus (T1DM), and type 2 diabetes mellitus (T2DM) using fractal analysis (FA) and histogram analysis (HA) on panoramic radiographs. While several studies [19,20] examined these conditions individually, to the best of our knowledge, none of these previous studies evaluated their effects using both FD and HA within a consistent anatomical framework. Given that endocrine disorders, such as thyroid diseases and diabetes, can influence bone metabolism and structure [2], assessing these changes is crucial for understanding their impact on bone health.

Radiologic imaging techniques play a key role in evaluating disease-related changes in bone structure. Panoramic radiographs are widely used due to their broad anatomical coverage and low radiation exposure. Non-invasive techniques like fractal analysis allow for the quantification of trabecular bone structure [13,14] and have been employed to evaluate bone alterations in systemic conditions such as diabetes [19], thalassemia [25], familial Mediterranean fever [26], sickle cell disease [27], osteogenesis imperfecta [28], chronic renal failure [29], hyperparathyroidism [30], and celiac disease [31]. Fractal dimension provides numerical assessments of trabecular complexity, with greater values reflecting increased architectural complexity [15]. Consequently, fractal analysis was deemed a suitable method for examining changes in jawbone architecture in our study.

Studies [13] have indicated that panoramic radiography is a reliable method for conducting fractal analysis. Nevertheless, there is currently no consensus regarding the gold standard among various radiographic techniques. Yavuz et al. [32] compared periapical, panoramic, and cone beam computed tomography (CBCT) images in evaluating trabecular bone structure using fractal analysis and found that periapical and panoramic radiographs produced comparable and consistent results, whereas CBCT images showed discrepancies and lacked correlation with the other modalities. Based on these findings, our study utilized panoramic radiographs for bone structure assessment. Orthopantomograms were selected for FD analysis due to their routine use in dental practice and their lower radiation exposure compared to CBCT.

Digital images are composed of pixels, each containing brightness and color data. Pixel intensity (PI) represents the grayscale value of a pixel, reflecting its degree of brightness or darkness. Histogram analysis (HA) allows for the numerical evaluation of pixel brightness variations within an image [17,18].

Both Type 1 Diabetes Mellitus (T1DM) and Type 2 Diabetes Mellitus (T2DM) are associated with impaired bone health due to a variety of metabolic disturbances. Chronic hyperglycemia disrupts osteoblast function and promotes the accumulation of advanced glycation end-products (AGEs), which compromise bone strength. Additionally, prolonged disease duration and poor glycemic control increase the risk of fractures. Lower levels of glucose-dependent insulinotropic peptide (GIP) and glucagon-like peptide-1 (GLP-1) reduce bone formation, while systemic inflammation accelerates bone resorption [4]. Given these complex metabolic effects, quantitative assessment of bone structure in diabetic patients is considered essential.

Despite this, our study did not reveal significant differences in fractal dimension (FD) or histogram analysis (HA) values between diabetic patients and healthy controls. This aligns with previous findings but also highlights methodological variations across literature. For instance, one study [19] evaluated mandibular cortical width (MCW), panoramic mandibular index (PMI), mandibular cortical index (MCI), and FD in panoramic radiographs of T1DM and T2DM patients. Although they reported reduced cortical measurements in T1DM patients, FD values (1.25 ± 0.10) did not significantly differ from those of the control group (1.29 ± 0.10). Similarly, Dedeoğlu et al. [33] performed fractal analysis on panoramic radiographs taken five years apart in T2DM patients and controls. While no group-based differences were found, they noted a time-dependent decline in FD values, suggesting progressive microarchitectural changes that may not be immediately evident in cross-sectional comparisons.

Other studies [34,35,36] have used alternative imaging techniques to assess bone quality in diabetic individuals. Ay et al. [34] employed DXA-calibrated copper step wedge phantoms on panoramic radiographs to estimate bone mineral density (BMD) in T2DM patients, again finding no significant difference compared to controls. Jolly et al. [35] used spiral computed tomography to compare trabecular and cortical bone densities in both jaws and observed no significant differences between well-controlled type 2 diabetes patients (HbA1c 6.1–8%) and non-diabetic individuals. In contrast, Kayıpmaz et al. [36] utilized cone beam computed tomography (CBCT) to examine FD in T2DM patients and reported no statistically significant difference in FD values between groups but noted thinner mandibular cortical bone in the diabetic cohort. These findings suggest that while FD may remain relatively stable, cortical bone thickness may be more sensitive to diabetic changes. A broader perspective is provided by Pan et al. [37], whose meta-analysis on T1DM and BMD revealed a significant reduction in total body BMD among T1DM patients. They also highlighted the potential modifying effects of age and gender on this relationship, suggesting a multifactorial influence of diabetes on bone metabolism.

Collectively, these findings reflect the heterogeneity of methodologies and patient populations studied and underscore the need for standardized protocols in assessing bone health in diabetic individuals. While FD analysis offers a non-invasive, reproducible metric for evaluating trabecular bone, it may not fully capture cortical alterations or systemic influences, which require complementary assessment methods.

Thyroid dysfunction has distinct effects on bone metabolism: hypothyroidism leads to reduced bone turnover due to impaired osteoid formation and mineralization, while hyperthyroidism increases bone resorption and formation, ultimately contributing to bone loss and fracture risk [8,38,39]. However, studies [8,40] have stated that these effects may vary depending on factors such as TSH level, gender, and age. In our study, fractal analysis (FA) revealed significantly lower FD values in hyperthyroid patients compared to controls specifically in the ROI1 region (the geometric center of the area between the mandibular notch and the mandibular foramen), with no significant differences observed in other regions. Our findings suggest that bone alterations in hyperthyroidism may be localized rather than generalized. This finding aligns partially with Öztürk et al. [20], who also used panoramic radiographs for fractal analysis and reported significant differences in ROI1(geometric center of the area between the mandibular notch and the mandibular foramen) and ROI2 (the geometric center of mandibular angle) when comparing thyroid disorder patients (hyperthyroidism patients, ROI1: 0894 ± 0.5, ROI2: 0.995 ± 0.11; hypothyroidism patients, ROI1: 0992 ± 0.6; ROI2: 1.005 ± 0.99) with healthy controls (ROI1: 1.032 ± 1.09, ROI2:1.0.76 ± 0.99), but no significant differences were found in more anterior or basal mandibular sites. Although Nair et al. [41] employed a different methodology—assessing radiomorphometric indices such as the mental index (MI), panoramic mandibular index (PMI), antegonial index (AGI), gonial index (GI) and mandibular cortical index (MCI)—they similarly found lower bone measurements in hypothyroid patients, with only the AGI showing statistical significance. These results suggest that certain mandibular regions may be more sensitive to systemic metabolic changes.

Additionally, when assessing bone density and architecture in thyroid disorders, the patient’s treatment status should be carefully considered. Yılmaz et al. [42] evaluated both fractal dimension (FD) and radiomorphometric indices in digital panoramic radiographs of hypothyroid patients undergoing levothyroxine therapy. Their findings revealed no significant differences compared to healthy controls, suggesting a potential stabilizing effect of hormone replacement treatment on bone structure. Vestergaard et al. [8] conducted that untreated hyperthyroidism leads to a significant reduction in BMD and an increased risk of fractures, particularly in older populations, while treatment can reverse these effects over time.

However, this study has several limitations. It did not account for variations in disease duration, severity, or glycemic control (e.g., HbA1c levels) in diabetic patients, which could influence bone metabolism and FD/HA values. Additionally, the potential effects of medications, including insulin, thyroid hormone replacement, and anti-diabetic drugs, on bone structure were not evaluated, which may have impacted the results. That said, the absence of these variables does not necessarily diminish the relevance of our findings. The study’s design aimed to minimize confounding factors by using a homogenous sample group, which likely reduced the potential impact of unmeasured variables on the overall findings. Future studies incorporating these additional variables will be essential for understanding the full spectrum of metabolic effects on bone health.

## 5. Conclusions

This study aimed to evaluate the impact of systemic conditions such as diabetes mellitus and thyroid dysfunction on mandibular bone structure using fractal dimension and histogram-based pixel intensity analyses on panoramic radiographs. While no significant differences in FD or HA values were observed between diabetic and control groups, hyperthyroid patients demonstrated significantly lower FD values in the ROI1 region, suggesting localized alterations in trabecular bone microarchitecture. These findings highlight the potential of fractal analysis (FA) as a non-invasive, quantitative tool for detecting subtle bone changes associated with systemic metabolic disorders. Fractal dimension (FD) and histogram analysis (HA) could be integrated into routine panoramic radiographs to help detect early bone changes in patients with systemic conditions like diabetes or thyroid disorders, supporting early diagnosis and personalized treatment in various dental fields. Future research should involve larger sample sizes and more comprehensive evaluations of disease duration, medication use, and biochemical markers of bone metabolism. To assess mandibular bone architecture more accurately in future studies, several advanced imaging modalities and techniques- such as High-Resolution Peripheral Quantitative Computed Tomography, micro-CT, Quantitative Ultrasound could be considered. Each of these offers distinct advantages that can complement or enhance the findings from traditional panoramic radiographs.

## Figures and Tables

**Table 1:** Distribution of gender and age among study groups.

		Study Groups												Chi-Square Test	
		Hyperthyroidism		Hypothyroidism		T1DM		T2DM		Controls		Total			
		*n*	%	*n*	%	*n*	%	*n*	%	*n*	%	*n*	%	X^2^	*p*
Gender	Male	9	22.5	6	15.0	13	32.5	9	22.5	9	22.5	46	23	3.5	0.478
	Female	31	77.5	34	85.0	27	67.5	31	77.5	31	77.5	154	77		
	Total	40	100	40	100	40	100	40	100	40	100	200	100		
Age														ANOVA	
														F	*p*
	Mean	50.83		50.78		50.85		50.78		50.73		50.79		0.001	1
	Median	52		49		52.5		51.5		49.5		51			
	Minimum	37.0		32.0		21.0		35.0		35.0		21			
	Maximum	66.0		72.0		75.0		74.0		74.0		75			
	Standard Deviation	8.1		8.6		13.5		9.0		9.9		9.9			

*n*, noun; T1DM, type 1 diabetes mellitus; T2DM, type 2 diabetes mellitus.

**Table 2:** Comparison of Fractal Dimension Values Between Patients with Endocrine Disorders (Hyperthyroidism, Hypothyroidism, Type 1 and Type 2 Diabetes Mellitus) and the Control Group.

	Hyperthyroidism	Control		Hypothyroidism	Control	
ROI	Mean ± SD	Mean ± SD	*p*	Mean ± SD	Mean ± SD	*p*
ROI1	1.406 ± 0.160	1.478 ± 0.157	0.018 ^a^	1.453 ± 0.158	1.478 ± 0.157	0.607 ^a^
ROI2	1.400 ± 0.152	1.461 ± 0.117	0.057 ^a^	1.453 ± 0.135	1.461 ± 0.117	0.651 ^a^
ROI3	1.510 ± 0.131	1.526 ± 0.105	0.969 ^a^	1.526 ± 0.070	1.526 ± 0.105	0.416 ^a^
ROI4	1.547 ± 0.057	1.541 ± 0.061	0.647 ^b^	1.554 ± 0.060	1.541 ± 0.061	0.316 ^b^
	Type 1 DM	Control		Type 2 DM	Control	
ROI1	1.498 ± 0.150	1.478 ± 0.157	0.408 ^a^	1.438 ± 0.180	1.478 ± 0.157	0.277 ^a^
ROI2	1.454 ± 0.131	1.461 ± 0.117	0.916 ^a^	1.453 ± 0.160	1.461 ± 0.117	0.795 ^a^
ROI3	1.533 ± 0.087	1.526 ± 0.105	0.988 ^a^	1.545 ± 0.062	1.526 ± 0.105	0.780 ^a^
ROI4	1.521 ± 0.087	1.541 ± 0.061	0.253 ^b^	1.543 ± 0.052	1.541 ± 0.061	0.765 ^a^

ROI, region of interest; SD, standart deviation; ^a^, Mann Whitney U; ^b^, *t*-test; DM, diabetes mellitus.

**Table 3:** Comparison of Histogram Analysis Values Between Patients with Endocrine Disorders (Hyperthyroidism, Hypothyroidism, Type 1 and Type 2 Diabetes Mellitus) and the Control Group.

	Hyperthyroidism	Control		Hypothyroidism	Control	
ROI	Mean ± SD	Mean ± SD	*p*	Mean ± SD	Mean ± SD	*p*
ROI1	95.988 ± 27.529	108.063 ± 32.056	0.075 ^b^	111.134 ± 29.653	108.063 ± 32.056	0.658 ^b^
ROI2	76.368 ± 29.770	79.843 ± 30.411	0.607 ^b^	86.448 ± 32.121	79.843 ± 30.411	0.348 ^b^
ROI3	89.683 ± 18.830	86.231 ± 18.349	0.409 ^b^	89.924 ± 16.307	86.231 ± 18.349	0.344 ^b^
ROI4	116.103 ± 21.520	111.702 ± 18.459	0.329 ^b^	107.222 ± 19.790	111.702 ± 18.459	0.298 ^b^
	Type 1 DM	Control		Type 2 DM	Control	
ROI1	109.568 ± 28.771	108.063 ± 32.056	0.826 ^b^	117.618 ± 29.570	108.063 ± 32.056	0.170 ^b^
ROI2	82.291 ± 27.352	79.843 ± 30.411	0.706 ^b^	89.150 ± 29.037	79.843 ± 30.411	0.166 ^b^
ROI3	91.934 ± 18.693	86.231 ± 18.349	0.172 ^b^	93.185 ± 18.584	86.231 ± 18.349	0.096 ^b^
ROI4	113.142 ± 24.282	111.702 ± 18.459	0.766 ^b^	107.877 ± 21.399	111.702 ± 18.459	0.395 ^b^

ROI, region of interest; SD, standart deviation; ^b^, *t*-test; DM, diabetes mellitus.

**Table 4:** Spearman Correlation Between Fractal Dimension and Histogram Values.

**Fractal Dimension**	**Histogram**		
	ROI1	Spearman Correlation	0.286
		*p*	<0.001
		N	200
	ROI2	Spearman Correlation	0.260
		*p*	<0.001
		N	200
	ROI3	Spearman Correlation	0.110
		*p*	0.122
		N	200
	ROI4	Spearman Correlation	0.117
		*p*	0.100
		N	200

ROI, region of interest; N, noun.

## Data Availability

Dataset available on request from the corresponding author.

## Abbreviations

The following abbreviations are used in this manuscript:

T1DM	Type 1 diabetes mellitus
T2DM	Type 2 diabetes mellitus
FD	Fractal dimension
HA	Histogram analysis
BMD	Bone mineral density
TSH	Thyroid stimulating hormone
DXA	Dual-energy X-ray absorptiometry
FA	Fractal analysis
FD	Fractal dimension
PI	Pixel intensity
TIFF	Tagged image file format
ROI	Region of interest
DM	Diabetes mellitus
CT	Computed tomography
CBCT	Cone beam computed tomography
AGE	Advanced glycation end-products
GIP	Glucose-dependent insulinotropic peptide
GLP-1	Glucagon-like peptide-1
MCW	Mandibular cortical width
PMI	Panoramic mandibular index
MCI	Mandibular cortical index
MI	Mental index
AGI	Antegonial index
GI	Gonial index
